# *Shigella sonnei* O-Antigen Inhibits Internalization, Vacuole Escape, and Inflammasome Activation

**DOI:** 10.1128/mBio.02654-19

**Published:** 2019-12-17

**Authors:** Jayne L. Watson, Julia Sanchez-Garrido, Philippa J. Goddard, Vincenzo Torraca, Serge Mostowy, Avinash R. Shenoy, Abigail Clements

**Affiliations:** a Department of Life Sciences, Imperial College London, London, United Kingdom; b Department of Infectious Disease, MRC Centre for Molecular Bacteriology and Infection, Imperial College London, London, United Kingdom; c Department of Infection Biology, London School of Hygiene and Tropical Medicine, London, United Kingdom; GSK Vaccines

**Keywords:** O-Antigen, *Shigella*, host-pathogen interactions, inflammasomes, macrophages

## Abstract

Two *Shigella* species, Shigella flexneri and Shigella sonnei, cause approximately 90% of bacterial dysentery worldwide. While S. flexneri is the dominant species in low-income countries, S. sonnei causes the majority of infections in middle- and high-income countries. S. flexneri is a prototypic cytosolic bacterium; once intracellular, it rapidly escapes the phagocytic vacuole and causes pyroptosis of macrophages, which is important for pathogenesis and bacterial spread. In contrast, little is known about the invasion, vacuole escape, and induction of pyroptosis during S. sonnei infection of macrophages. We demonstrate here that S. sonnei causes substantially less pyroptosis in human primary monocyte-derived macrophages and THP1 cells. This is due to reduced bacterial uptake and lower relative vacuole escape, which results in fewer cytosolic S. sonnei and hence reduced activation of caspase-1 inflammasomes. Mechanistically, the O-antigen (O-Ag), which in S. sonnei is contained in both the lipopolysaccharide and the capsule, was responsible for reduced uptake and the type 3 secretion system (T3SS) was required for vacuole escape. Our findings suggest that S. sonnei has adapted to an extracellular lifestyle by incorporating multiple layers of O-Ag onto its surface compared to other *Shigella* species.

## Abstract (precis)

Diarrheal disease remains the second leading cause of death in children under five. Shigella remains a significant cause of diarrheal disease with two species, S. flexneri and S. sonnei, causing the majority of infections. S. flexneri are well known to cause cell death in macrophages, which contributes to the inflammatory nature of *Shigella* diarrhea. Here, we demonstrate that S. sonnei causes less cell death than S. flexneri due to a reduced number of bacteria present in the cell cytosol. We identify the O-Ag polysaccharide which, uniquely among *Shigella* spp., is present in two forms on the bacterial cell surface as the bacterial factor responsible. Our data indicate that S. sonnei differs from S. flexneri in key aspects of infection and that more attention should be given to characterization of S. sonnei infection.

## INTRODUCTION

*Shigella* are the causative agents of shigellosis, infecting an estimated 125 million people annually. Children under five are most at risk with a third of all deaths due to *Shigella* occurring among this age group (1). Closely related to Escherichia coli, the genus is made up of four species; Shigella flexneri, S. sonnei, S. dysenteriae, and S. boydii. These species are divided into serotypes based on the O-antigen (O-Ag) structure. S. flexneri and S. sonnei are responsible for the majority of infections; however, dominance is highly dependent on the socioeconomic status of an area. S. flexneri is associated with poor water sanitation and hygiene in developing countries. In sub-Saharan Africa and Asia, S. flexneri accounts for 66% of cases and S. sonnei 24% of cases (2). However, in areas with good socioeconomic conditions and a high gross domestic product per capita, such as North America and Europe, S. sonnei is responsible for up to 80% of infections (3). Transitional countries that have recently undergone socioeconomic improvements show a shift from S. flexneri to S. sonnei as the dominant species (4–6). As a number of large populous countries undergo this shift (e.g., Brazil, India, and China), S. sonnei is emerging as an important pathogen.

The pathogenesis of S. sonnei is poorly understood and generally assumed to be similar to S. flexneri. The growing importance of S. sonnei has led to a reevaluation of its pathogenesis and has revealed some important differences from S. flexneri. These include a novel adhesin (7, 8), an antibacterial type 6 secretion system (T6SS) (9), and a group 4 capsule (G4C), which protects it from serum-mediated killing (10). Both species have a homologous type 3 secretion system (T3SS) that promotes secretion of effectors into host cells.

Unlike other *Shigella* species which contain multiple serotypes, there is only one S. sonnei serotype. The genes encoding biosynthesis and export of the O-Ag are encoded on the pSS virulence plasmid and were horizontally acquired from Plesiomonas shigelloides. In all other *Shigella* spp., these genes are located on the chromosome (11). S. sonnei O-Ag is composed of two unusual sugars, 2-acetamido-2-deoxy-l-altruronic acid and 2-acetamido-2-deoxy-l-fucose, which are not present in the O-Ags of other *Shigella* spp. or indeed in many bacteria (12). Importantly, the G4C of S. sonnei is also composed of the O-Ag polysaccharide, linked to an unknown lipid anchor rather than the lipid A/core as in the lipopolysaccharide (LPS) (10). Therefore, the surface of S. sonnei is covered with two O-Ag layers.

Pyroptotic cell death is considered an important component of S. flexneri pathogenesis (13), allowing S. flexneri to escape macrophage-mediated killing, induce local inflammation, and invade epithelial cells from the basolateral side (14). In the canonical pathway for caspase-1 activation and pyroptosis, NOD and leucine-rich repeat containing proteins with CARD or PYD (NLRCs or NLRPs), AIM2-like receptors or Pyrin protein can respond to pathogen- and/or danger-associated molecular patterns. This leads to the assembly of the sensor, e.g., NLRP3 or NLRC4, and the adaptor protein, ASC, into a signaling platform, known as the inflammasome, which activates caspase-1 (15). In the noncanonical pathway, caspase-4 directly senses and is activated by cytosolic LPS (16). Active caspase-1 and active caspase-4 can cleave gasdermin-D (GSDMD) (17). Once cleaved, the N-terminal of GSDMD forms pores in the cell membrane to cause swelling and membrane rupture. The proinflammatory cytokines interleukin-1β (IL-1β) and IL-18 are also cleaved by active caspase-1 into their mature forms and released (18, 19).

S. flexneri can activate the NLRC4 and NLRP3 inflammasomes (20). The T3SS needle and rod proteins (MxiH and MxiI, respectively) are recognized by hNaip/mNaip1 and mNaip2 proteins, which interact with NLRC4 and promote caspase-1 activation (21, 22). NLRP3 senses decreased cytosolic potassium levels and activates caspase-1 (23). A T3SS effector, IpaH7.8, has been shown to be important for activation of both the NLRC4 and NLRP3 inflammasomes (20). In the case of *Shigella*, it is unclear whether pyroptosis benefits the host or the bacteria. S. flexneri is thought to use pyroptosis to escape the macrophage and infect epithelial cells. However, recent studies using *Salmonella* suggest that pyroptosis results in killing of bacteria by forming pore-induced intracellular traps (24) or GSDMD targeting of bacterial membranes (25). It is currently unknown whether S. sonnei activates the same inflammasomes as S. flexneri and whether this is beneficial for the host or bacteria.

In this study, we demonstrate for the first time that S. sonnei induces caspase-1-dependent pyroptosis of human macrophages. However, we observed that equivalent bacterial inocula induced much less cell death for S. sonnei than S. flexneri. We show this is due to the O-Ag of S. sonnei, which reduces internalization and vacuole escape, resulting in less cytosolic bacteria. Our studies reveal an important role for the S. sonnei O-Ag in regulating bacterial interactions with macrophages, with one consequence being a reduction in inflammatory cell death.

## RESULTS

### *S. sonnei* induces less macrophage cell death than *S. flexneri.*

**FIG 1:**
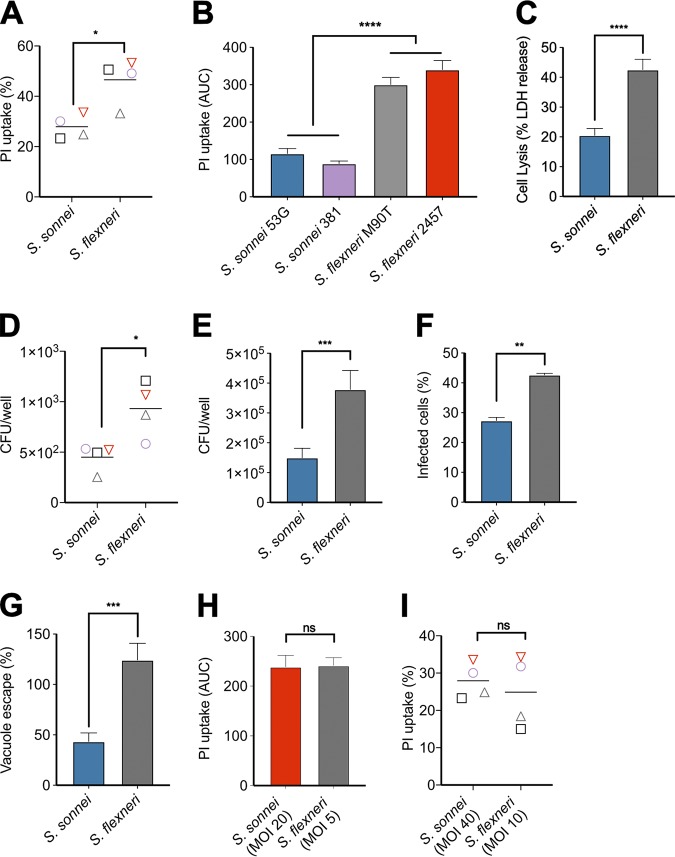
S. sonnei induces less pyroptosis of macrophages than S. flexneri. (A) Primary hMDMs were infected with the indicated *Shigella* strains, and cell death was measured by PI uptake at 3 h postinfection. *, *P* < 0.05 (by paired Student *t* test; *n* = 4 independent repeats from two donors). (B and C) THP1 cells were infected with the indicated WT *Shigella* strains. Cell death was measured by PI uptake over a 3-h time course and is plotted as the area under the curve (AUC) (*n* = 3) (B) or LDH release at 3 h postinfection (*n* = 9) (C). ****, *P* < 0.0001 (by one-way ANOVA with Tukey’s multiple-comparison test) (B) or ****, *P* < 0.0001 (by paired Student *t* test (C). (D) hMDMs and (E) THP1 cells were infected with S. sonnei or S. flexneri and gentamicin-protected intracellular bacteria determined by CFU enumeration. (D) *, *P* < 0.05 (by paired Student *t* test, *n* = 4 independent repeats from two donors) and (E) ***, *P* < 0.001 by paired Student *t* test (*n* = 11). (F) Immunofluorescence microscopy was used to visualize intracellular/extracellular bacteria, and the percentage of infected THP1 cells was calculated. **, *P* < 0.01 (by paired Student *t* test, *n* = 3). (G) THP1 cells were infected with S. sonnei or S. flexneri and subsequently treated with gentamicin alone or gentamicin/chloroquine to determine the percentage of cytosolic bacteria that have escaped the vacuole. ***, *P* < 0.001 (by paired Student *t* test, *n* = 4). (H) THP1 cells were infected with S. sonnei at an MOI of 20 or with S. flexneri at an MOI of 5. Cell death was measured by PI uptake over a 3-h time course and is plotted as the AUC. ns, nonsignificant (by paired Student *t* test, *n* = 3). (I) hMDMs were infected with S. sonnei at an MOI of 40 or with S. flexneri at an MOI of 10, and the PI uptake was measured at 3 h postinfection. ns, nonsignificant (by paired Student *t* test, *n* = 4 independent repeats from two donors).

Previous research into the interactions of *Shigella* with macrophages has largely focused on S. flexneri, which robustly induces pyroptosis in macrophages (20). To investigate whether S. sonnei behaved in a similar manner, we infected primary human CD14^+^ monocyte-derived macrophages (hMDMs) and measured the uptake of propidium iodide (PI), as an indicator of membrane damage that precedes pyroptosis. Unexpectedly, S. sonnei induced 50% less PI uptake than S. flexneri (Fig. 1A).

Similar experiments in phorbol-12-myristate-13-acetate (PMA)-differentiated THP1 cells recapitulated the reduced PI uptake during S. sonnei infection compared to S. flexneri (Fig. 1B). In addition, a lactate dehydrogenase (LDH) release assay comparing lytic cell death showed S. sonnei induced less cell death than S. flexneri (Fig. 1C). To ensure the reduced cell death was not a unique feature of the widely used S. sonnei strain 53G, we included a recent clinical isolate, S. sonnei 381, alongside S. sonnei 53G and compared these to two different S. flexneri strains M90T (serotype 5a) and 2457T (serotype 2a). Notably, both S. sonnei strains induced lower PI uptake in macrophages (Fig. 1B).

### There are fewer cytosolic *S. sonnei* than *S. flexneri*.

Induction of macrophage cell death by S. flexneri requires the bacteria to be cytosolic, which entails two steps: internalization and vacuole escape. We hypothesized that differences in these processes between S. flexneri and S. sonnei might be responsible for the differences in cell death observed. To investigate why S. sonnei induced less cell death, we treated hMDMs or PMA-treated THP1 cells with 50 μM Z-VAD-fmk, a pan-caspase inhibitor, to inhibit cell death (Fig. S1A in the supplemental material) and performed a gentamicin protection assay to calculate the number of intracellular bacteria (Fig. 1D and E). S. sonnei-infected macrophages had reduced numbers of intracellular bacteria compared to S. flexneri.

10.1128/mBio.02654-19.1 FIG S1 Characterization of cell death in *Shigella* infected THP1 cells. (A) THP1 cells were left untreated or treated with 50 μM Z-VAD-fmk one hour prior to infection with S. flexneri. Cell death was measured by PI uptake over a 3 h time-course and plotted as the AUC. ****, *P* < 0.0001 (by unpaired Student t test, *n* = 3). (B) THP1 cells were infected with the indicated MOIs of S. sonnei or S. flexneri, and the numbers of cytosolic CFU were enumerated after treating cells with gentamicin and chloroquine to kill extracellular and vacuolar bacteria. ns, nonsignificant (by one-way ANOVA, *n* = 4). (C) THP1 cells were infected with wild-type S. sonnei and S. flexneri and their respective T3SS mutants. Cell death was measured by LDH release at 3 h. **, *P* < 0.01; ***, *P* < 0.001; ****, *P* < 0.0001 (by one-way ANOVA, *n* = 3). (D) THP1 cells were infected with S. sonnei or S. flexneri and the respective T3SS mutants. Cell death was measured by LDH release at 3 h. ns, nonsignificant (by one-way ANOVA, *n* = 4). Download FIG S1, TIF file, 1.5 MB. Copyright © 2019 Watson et al. 2019 Watson et al. This content is distributed under the terms of the Creative Commons Attribution 4.0 International license.

As the earliest time point that can be measured in the gentamicin protection assay is 1 h 40 min postinfection, it is possible bacteria were already killed by this time point, which would misrepresent the relative efficiency of internalization (because internalized and killed bacteria would not be detected). To address this, we enumerated intracellular bacteria by differential staining at 40 min postinfection, which confirmed that fewer THP1 cells harbored intracellular bacteria when infected with S. sonnei than when infected with S. flexneri (Fig. 1F).

Internalized S. flexneri rapidly lyse the phagocytic/endosomal vacuole in order to access the cell cytosol and escape lysosomal degradation (26). To investigate how well S. sonnei escaped into the cytosol, we used chloroquine, an antibiotic that only accumulates in vacuoles at high enough concentrations to kill bacteria, allowing discrimination between cytosolic and vacuolar bacteria (27). S. sonnei showed a reduction in vacuole escape compared to S. flexneri (Fig. 1G). Taken together, these data indicated there are less cytosolic S. sonnei compared to S. flexneri at the same multiplicity of infection (MOI), which may result in the reduced macrophage cell death observed with S. sonnei. By increasing the S. sonnei MOI to obtain equivalent numbers of cytosolic bacteria to S. flexneri (Fig. S1B), S. sonnei and S. flexneri induced similar levels of cell death (Fig. 1H and J) and cell lysis (Fig. S1C). These findings confirm that cytosolic bacteria are required for induction of cell death in S. sonnei and S. flexneri and that S. sonnei does not access the cytosol as efficiently as S. flexneri.

### The T3SS is required for vacuole escape but not internalization of *S. sonnei*.

**FIG 2:**
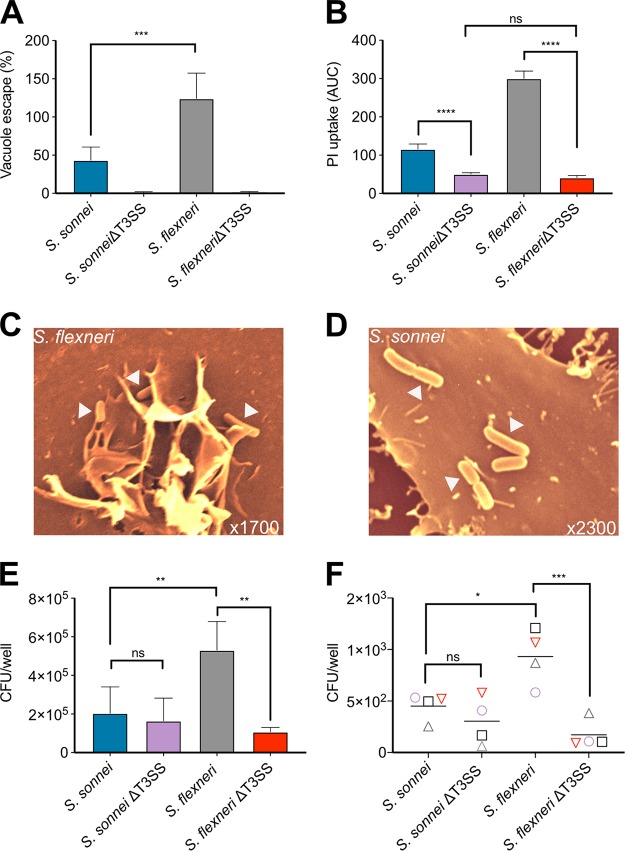
For S. sonnei, vacuole escape and cell death are T3SS dependent, but internalization is T3SS independent. (A) THP1 cells were infected with S. sonnei or S. flexneri and their respective T3SS mutants and subsequently treated with gentamicin alone or gentamicin/chloroquine to determine the percentage of cytosolic bacteria that escaped the vacuole. ***, *P* < 0.001 (by one-way ANOVA with Tukey’s multiple-comparison test, *n* = 4). (B) THP1 cells were infected with WT S. sonnei and S. flexneri and their respective T3SS mutants. Cell death was measured by PI uptake over a 3-h time course and is plotted as the AUC. ns, nonsignificant; ****, *P* < 0.0001 (by one-way ANOVA with Tukey’s multiple-comparison test, *n* = 3). (C and D) HeLa cells were infected with WT S. sonnei and S. flexneri for 10 min before being washed and fixed for SEM analysis. Arrows indicate bacteria attached to the cell surface. THP1 cells (E) and hMDMs (F) were infected with WT or T3SS-deficient S. sonnei and S. flexneri, and gentamicin-protected internalized bacteria were determined by CFU enumeration. ns, nonsignificant; *, *P* < 0.05; **, *P* < 0.01 (by one-way ANOVA with Tukey’s multiple-comparison test, *n* = 4 [E] and *n* = 4 [F] independent repeats from two donors).

The T3SS of S. flexneri is required for bacteria to lyse the phagocytic vacuole and access the cytosol (28). Consistent with this, a S. sonnei T3SS mutant (Δ*mxiD*) had an impaired ability to escape the vacuole (Fig. 2A) and reduced cell death measured by PI uptake (Fig. 2B) and LDH release (Fig. S1D). The S. sonnei T3SS was required to induce vacuole lysis and hence produce cytosolic bacteria.

It is unclear whether *Shigella* internalization into macrophages is predominantly T3SS-dependent invasion or phagocytic uptake. T3SS-mediated invasion of epithelial cells by S. flexneri triggers extensive membrane recruitment to engulf the bacteria. To visualize S. flexneri and S. sonnei uptake, we performed scanning electron microscopy (SEM) on infected cells and were able to see membrane recruitment around attached S. flexneri but not S. sonnei (Fig. 2C and D). Since phagocytic uptake and T3SS-mediated invasion both involve membrane rearrangement, these would be difficult to distinguish visually. Instead, we performed gentamicin protection assays with wild-type (WT) and T3SS mutant strains to quantify the number of intracellular bacteria in macrophages. Our experiments showed that in both hMDMs and THP1 cells, internalization into macrophages was T3SS dependent for S. flexneri but not S. sonnei (Fig. 2E and F). This suggested that the majority of S. flexneri actively invaded macrophages, in contrast to S. sonnei, which were mainly internalized by phagocytic uptake.

### *S. sonnei* and *S. flexneri* induce similar pyroptosis pathways in infected macrophages.

**FIG 3:**
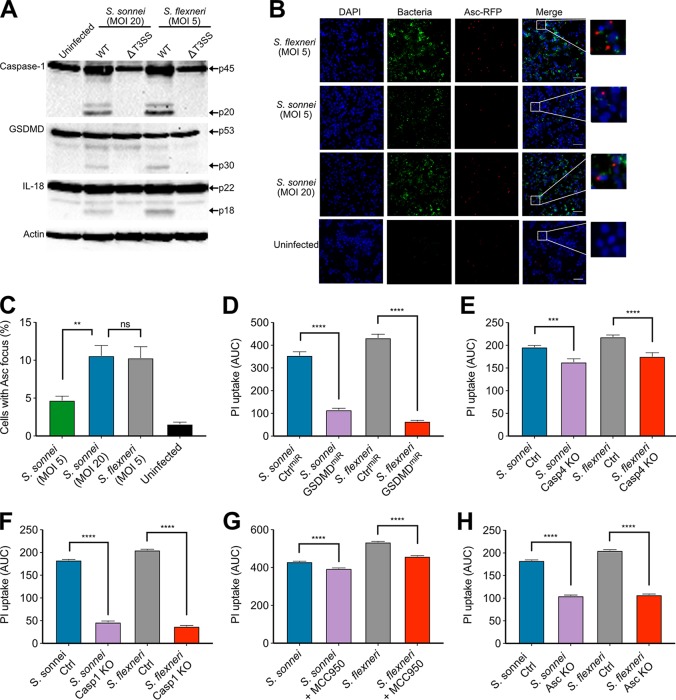
S. sonnei and S. flexneri induce similar pyroptosis pathways when normalized for numbers of cytosolic bacteria. (A) Immunoblots were performed on *Shigella-*infected THP1 cells to visualize cleavage of caspase-1, GSDMD, and IL-18 at 3 h postinfection. (B and C) ASC^mRFP^ THP1 cells were infected with fluorescent *Shigella* (green) at the indicated MOIs and, at 3 h postinfection, the ASC focus formation was visualized (red). DAPI staining was used to visualize DNA (blue). Representative micrographs for each strain are shown. The area indicated by the white box in the merged panel is enlarged to show single ASC foci in infected cells and the lack of ASC foci in uninfected cells. ASC focus formation was enumerated in panel C. ns, nonsignificant; **, *P* < 0.01 (by one-way ANOVA, *n* = 4). (D) THP1 cells expressing nontargeting miRNA or GSDMD-targeting miRNA were infected with S. sonnei (MOI of 20) or S. flexneri (MOI of 5) for 3 h. Cell death was measured by PI uptake over a 3-h time course and is plotted as the AUC. ****, *P* < 0.0001 (by one-way ANOVA with Tukey’s multiple-comparison test, *n* = 3). (E) Control THP1 cells (Ctrl) and THP1 cells deficient for caspase-4 (Casp4 KO) were infected with S. sonnei (MOI of 20) or S. flexneri (MOI of 5). Cell death was measured by PI uptake over a 3-h time course and is plotted as the AUC. ***, *P* < 0.001; ****, *P* < 0.0001 (by one-way ANOVA with Tukey’s multiple-comparison test, *n* = 3). (F) Control THP1 cells (Ctrl) and THP1 cells deficient for caspase-1 (Casp1 KO) were infected with S. sonnei (MOI of 20) or S. flexneri (MOI of 5). Cell death was measured by PI uptake over a 3-h time course and is plotted as the AUC. ****, *P* < 0.0001 (by one-way ANOVA with Tukey’s multiple-comparison test, *n* = 3). (G) THP1 cells left untreated or treated with 5 μM MCC950 were infected with S. sonnei (MOI of 20) or S. flexneri (MOI of 5) for 3 h. Cell death was measured by PI uptake over a 3-h time course and is plotted as the AUC. ****, *P* < 0.0001 (by one-way ANOVA with Tukey’s multiple-comparison test, *n* = 4). (H) THP1 cells and ASC-deficient THP1 cells were infected with S. sonnei (MOI of 20) or S. flexneri (MOI of 5) for 3 h. Cell death was measured by PI uptake over a 3-h time course and is plotted as the AUC. ****, *P* < 0.0001 (by one-way ANOVA Tukey’s multiple-comparison test, *n* = 3).

Given that cytosolic bacteria induce cell death through inflammasome activation, we characterized the inflammasome pathways activated by S. sonnei. Since the S. flexneri inflammasome activators MxiH, MxiI, and IpaH7.8 proteins are 100% identical between S. sonnei 53G and S. flexneri M90T, we hypothesized they would activate the NLRC4 inflammasome. At comparable levels of cytosolic bacteria, similar activation of caspase-1 and proteolytic cleavage of GSDMD and IL-18 were observed (Fig. 3A). The involvement of the inflammasome pathway was confirmed using ASC^mRFP^ THP1 cells, which revealed that both bacteria induced comparable levels of cells with ASC-containing inflammasome foci during infection (Fig. 3B and C). Further, infected GSDMD-silenced THP1 cells (THP^GSDMD-miR^, validated in Fig. S2A and B) underwent reduced cell death, suggesting pyroptosis is the dominant type of cell death induced by S. sonnei and S. flexneri (Fig. 3D).

10.1128/mBio.02654-19.2 FIG S2 Phenotypic confirmation of THP1 cell lines. (A) GSDMD^miR^ and Control^miR^ THP1 cells were treated with LPS and nigericin. Cell death was measured by PI uptake over a 3 h time-course and plotted as the AUC. ****, *P* < 0.0001 (by one-way ANOVA, *n* = 3). (B) Representative immunoblots show silencing of GSDMD, and β-actin serves as a loading control. (C and D) Control THP1 cells and THP1 cells deficient for caspase-1, ASC, or caspase-4 were transfected with LPS to induce caspase-4 activation (C) or LPS plus nigericin to induce canonical NLRP3 activation (D). Cell death was measured by PI uptake over a 3-h time course and is plotted as the AUC. ****, *P* < 0.0001 (by one-way ANOVA, *n* = 3). (E) Representative immunoblot shows knockout of caspase-1, ASC, or caspase-4 loss in the appropriate cell line. β-Actin serves as a loading control. (F) LPS plus nigericin was added to THP1 cells incubated with or without 5 μM MCC950. Cell death was measured by PI uptake over a 3-h time course and is plotted as the AUC. ****, *P* < 0.0001 (by one-way ANOVA, *n* = 4). Download FIG S2, TIF file, 1.5 MB. Copyright © 2019 Watson et al. 2019 Watson et al. This content is distributed under the terms of the Creative Commons Attribution 4.0 International license.

Cells deficient in caspase-4 showed reduced pyroptosis (Fig. 3E), however, loss of caspase-1 almost completely abolished pyroptosis (Fig. 3F; all knockout cells are validated in Fig. S2C to E), indicating the canonical pathway of pyroptosis predominates in S. sonnei- and S. flexneri-infected macrophages. Treatment with the NLRP3 inhibitor, MCC950 (29), did not markedly affect cell death (Fig. 3G and validated in Fig. S2F), suggesting that NLRP3 plays a minor role in pyroptosis. ASC-deficient THP1 cells showed a partial reduction in cell death levels compared to WT THP1 cells (Fig. 3H). Taken together, these results are consistent with NLRC4 activation contributing to pyroptosis during S. sonnei infection of human macrophages, which is similar to previous reports for S. flexneri.

### The T6SS and LVP instability do not account for reduced cell death caused by *S. sonnei*.

**FIG 4:**
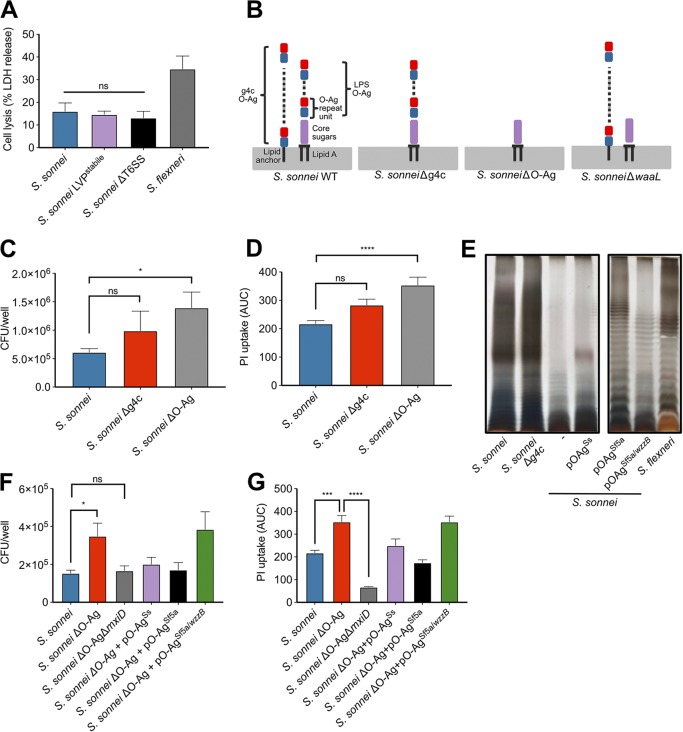
The presence of S. sonnei O-Ag reduces cell death and internalization. (A) THP1 cells were infected with S. sonnei WT, LVP^Stabile^, or ΔT6SS strains, and cell death was measured by LDH release at 3 h postinfection. ns, nonsignificant (by one-way ANOVA with Tukey’s multiple-comparison test, *n* = 3). (B) S. sonnei has O-Ag attached to the cell surface in two different forms, as the conventional O-Ag attached to the Lipid A-core of the LPS and as an O-Ag capsule attached to the cell surface by an unknown lipid anchor. S. sonnei ΔG4C has only O-Ag incorporated into the LPS, S. sonnei ΔO-Ag has neither G4C or LPS O-Ag, and S. sonnei *ΔwaaL* has only G4C O-Ag. (C) THP1 cells were infected with S. sonnei WT, ΔG4C, or ΔO-Ag strains, and the gentamicin-protected internalized bacteria were determined by CFU enumeration. *, *P* < 0.05 (by one-way ANOVA with a mixed-effect model with Sidak’s multiple-comparison test, *n* = 10 [wild type] and 7 [mutants]). (D) THP1 cells were infected with S. sonnei WT, ΔG4C, or O-Ag strains. Cell death was measured by PI uptake over a 3-h time course and is plotted as the AUC. ****, *P* < 0.0001 (by one-way ANOVA with Tukey’s multiple-comparison test, *n* = 4). (E) Crude LPS was purified from the indicated S. sonnei and S. flexneri strains, separated by 12% SDS-PAGE, and visualized by a modified silver stain. (F) THP1 cells were infected with S. sonnei WT, S. sonnei ΔO-Ag strain, or the S. sonnei ΔO-Ag strain complemented with S. sonnei O-Ag (pO-Ag^Ss^), the S. flexneri O-Ag (pO-Ag^Sf5a^), or the S. flexneri O-Ag, and WzzB (pO-Ag^Sf5a/wzzB^), and gentamicin-protected internalized bacteria were determined by CFU enumeration. *, *P* < 0.05; **, *P* < 0.01 (by one-way ANOVA with Tukey’s multiple-comparison test, *n* = 4). (G) THP1 cells were infected with S. sonnei WT, S. sonnei ΔO-Ag, S. sonnei ΔO-Ag plus pO-Ag^Ss^, or S. sonnei ΔO-Ag plus pO-Ag^Sf5a^. Cell death was measured by PI uptake over a 3-h time course and is plotted as the AUC. ****, *P* < 0.0001 (by one-way ANOVA with Tukey’s multiple-comparison test, *n* = 4).

All *Shigella* spp. harbor a large virulence plasmid (LVP) that encodes the T3SS, its effectors and additional important virulence factors. The LVP of S. sonnei is less stable than S. flexneri due to the evolution of different toxin-antitoxin systems (30). We inserted an antibiotic resistance cassette onto the LVP to create a stabilized LVP and used this strain to test whether LVP loss affected the amount of cell death that was induced. The LVP stabilized S. sonnei induced similar cell lysis as WT S. sonnei, indicating that differences in plasmid retention was not responsible for the altered interaction with macrophages (Fig. 4A).

Even though the T6SS of S. sonnei has only been described to have antibacterial activity (9), T6SSs from other bacteria (e.g., Francisella tularensis [31, 32]) have activity within macrophages. We therefore created a S. sonnei T6SS mutant (Δ*tssB*) to determine whether there was any contribution by the T6SS to cell death but found no difference in LDH release (Fig. 4A), indicating that the T6SS was not responsible for the altered interaction with macrophages. Altogether, these results ruled out the loss of LVP or a contribution by the T6SS in the reduced cell death observed for S. sonnei.

### *S. sonnei* O-Ag prevents internalization into macrophages.

S. sonnei O-Ag is incorporated into the G4C as well as being attached to the lipid A/core of LPS (Fig. 4B). The incorporation of the O-Ag into LPS and G4C is genetically separable, which we exploited to investigate their respective roles in the interaction with macrophages. The G4C of S. sonnei reduces bacterial invasion of epithelial cells by impairing T3SS activity (10) and could therefore play a similar role in macrophage internalization. We confirmed that S. sonnei ΔG4C invaded HeLa cells more efficiently (see Fig. S3B). Uptake and pyroptosis induced by S. sonnei ΔG4C was statistically similar to WT bacteria, although we did observe slightly greater cell death with the ΔG4C mutant (Fig. 4C and D). This was consistent with predominantly phagocytic uptake of S. sonnei by macrophages.

10.1128/mBio.02654-19.3 FIG S3 Characterization of S. sonnei O-Ag mutants. (A) HeLa cells were infected with S. sonnei, S. sonnei ΔG4C, or S. flexneri, and gentamicin-protected internalized bacteria were determined by CFU enumeration. *, *P* < 0.05 (by one-way ANOVA, *n* = 3). (B) THP1 cells were infected with WT S. sonnei or S. sonnei Δ*waaL* strains, and gentamicin-protected internalized bacteria were determined by CFU enumeration. ns, nonsignificant (by one-way ANOVA, *n* = 5). (C and D) Crude LPS was purified from the indicated S. sonnei and S. flexneri strains, and the presence of the S. sonnei O-Ag (C) or S. flexneri 5a O-Ag (D) was detected using serotype-specific antibodies. Download FIG S3, TIF file, 1.5 MB. Copyright © 2019 Watson et al. 2019 Watson et al. This content is distributed under the terms of the Creative Commons Attribution 4.0 International license.

We then deleted the O-Ag synthesis operon (genes *wbgT* to *wbgZ*) (33) to create a strain devoid of all O-Ag (both LPS and G4C linked) (Fig. 4B). This strain (ΔO-Ag) demonstrated increased internalization and cell death compared to S. sonnei Δg4c (Fig. 4C and D) and WT S. sonnei. In contrast, an LPS O-Ag-deficient strain (Δ*waaL*), which retains the G4C, showed equivalent internalization as WT S. sonnei (see Fig. S3A). Therefore, the presence of the S. sonnei O-Ag *per se*, rather than specifically the O-Ag in the capsule or attached to the lipid A/core, impedes macrophage internalization, and its complete removal enhances bacterial internalization.

We have shown that S. sonnei cell death is T3SS dependent due to the requirement for cytosolic bacteria. The T3SS tip accessibility has previously been shown to be enhanced upon removal of the G4C and further exposed by removal of the O-Ag (10). We therefore hypothesized that the O-Ag was impeding T3SS-mediated invasion. To test this, we created a T3SS mutant in the O-Ag-deficient strain (ΔO-AgΔ*mxiD*). In keeping with our hypothesis, this strain had wild-type levels of internalization (Fig. 4E) but impaired activation of cell death because it is unable to escape the vacuole. To further investigate the role of the O-Ag in shielding the T3SS, we complemented the O-Ag mutant (ΔO-Ag) with either the S. sonnei O-Ag synthesis operon (pSS) or the S. flexneri 5a O-Ag synthesis and modification operons (pSf5a) (34–36) (Fig. 4E). Both complemented strains impeded internalization of S. sonnei (Fig. 4F) and, as a consequence, reduced the level of cell death similar to those observed with WT S. sonnei (Fig. 4F). Interestingly, complementation with pSf5a produced a S. flexneri-like O-Ag ladder that migrated differently on SDS-PAGE than when expressed in S. flexneri. To determine whether this was due to different modal length of O-Ag controlled by WzzB, we introduced the *wzzB*^Sf^ onto the pSf5a complementation plasmid (pSf5a/*wzzB*). In this strain the modal length of the O-Ag resembled that of the WT S. flexneri; however, a low level of expression was observed. The levels of internalization and cell death were not reduced to the levels of the WT S. sonnei and instead resembled the levels of the O-Ag mutant.

## DISCUSSION

S. flexneri is known to induce pyroptosis in macrophages. This is considered a key step in the pathogenesis of *Shigella* since it allows bacteria to infect epithelial cells from the preferred basolateral side and leads to bacterial dissemination. In addition, pyroptosis creates an inflammatory response causing the recruitment of neutrophils, which disrupts the epithelial cell barrier and allows more *Shigella* to traverse the epithelial layer (37).

**FIG 5:**
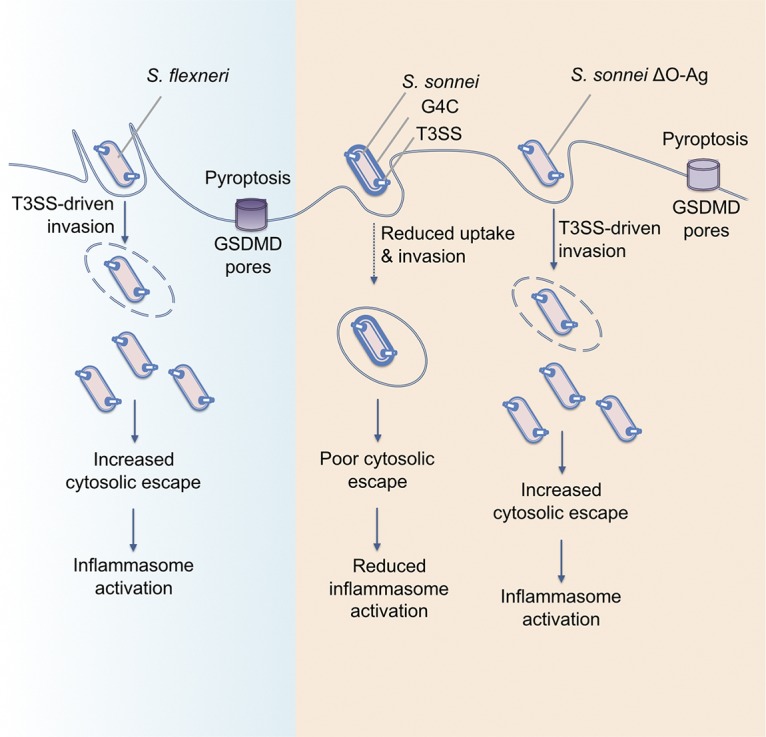
Model showing the interaction of S. flexneri, S. sonnei, and S. sonnei ΔO-Ag with macrophages. S. flexneri uses T3SS-mediated invasion and vacuole rupture to reach the cytosol, where it induces inflammasome activation, GSDMD pores, and pyroptosis. S. sonnei does not invade macrophages using its T3SS and only becomes intracellular through phagocytosis. Combined with a decreased ability to escape the vacuole, this reduces the number of cytosolic bacteria and leads to reduced inflammasome activation. The T3SS becomes accessible in S. sonnei ΔO-Ag, allowing the bacteria to invade macrophages and reach the cytosol, where they can activate the inflammasome and cause pyroptosis.

Here, we present evidence that S. sonnei does not use the same mechanisms during infection as S. flexneri (summarized in Fig. 5). In line with previous reports, we found that S. flexneri induces rapid pyroptosis upon internalization of infected macrophages (20, 22). However, S. sonnei induced markedly less macrophage cell death, which was the result of a decreased number of cytosolic bacteria through a combination of fewer internalized S. sonnei and impaired vacuole escape. The requirement for cytosolic bacteria in the induction of inflammasomes was consistent for both S. sonnei and S. flexneri. Additional host responses are also likely to be affected by the reduced number of cytosolic bacteria for S. sonnei compared to S. flexneri.

Once S. sonnei and S. flexneri cytosolic numbers were normalized, pyroptosis proceeded via similar pathways and to similar levels. For both species, cell death was predominantly dependent on GSDMD and caspase-1, indicating the canonical inflammasome pathway is induced by *Shigella.* There may be a minor contribution to cell death for the noncanonical pathway, since immunoblots indicated that caspase-4 was activated by infection by both S. sonnei and S. flexneri and that caspase-4 deficiency or NLRP3 inhibition led to less pyroptosis over time than control cells. However, this difference was minor compared to that observed for ASC or caspase-1-deficient cells. NLRC4 has a caspase-recruitment and activation domain (CARD), which can enable its interaction with and activation of caspase-1 directly, bypassing the need for ASC (38–40). This suggests that the NLRC4 inflammasome has a prominent role in the cell death of S. sonnei-infected THP1 cells. These results are in line with those shown previously for S. flexneri, which suggest both the NLRP3 and NLRC4 inflammasomes are involved in S. flexneri-mediated macrophage death (20).

Interestingly, S. sonnei was able to reduce internalization into macrophages in an O-Ag-dependent manner. The O-Ag contributes to host immune evasion, and its role in evasion of complement mediated killing is well characterized (41). There are also examples of the O-Ag affecting cellular interactions, including impeding recognition and internalization by epithelial cells (*Salmonella* Typhimurium [42]) and macrophages (Burkholderia cenocepacia [43]). The modal length of the O-Ag from *Salmonella* Typhimurium or S. flexneri serotype 2a is important for T3SS-mediated invasion into macrophages and epithelial cells, respectively (44). Similarly, glucosylation of the S. flexneri serotype 5a O-Ag, which reduces the O-Ag length by half, enhances its invasiveness (45).

Unexpectedly, in our study the internalization of S. sonnei into macrophages was independent of its T3SS. This is in contrast to S. flexneri, which exhibits significant T3SS-mediated invasion into macrophages. This suggests that macrophage internalization is a combination of bacterium-driven invasion and phagocytic uptake for S. flexneri, whereas S. sonnei internalization is almost exclusively due to phagocytic uptake. S. sonnei O-Ag is incorporated into both the G4C and the LPS of S. sonnei. Only when all of the O-Ag layers of S. sonnei are removed can S. sonnei efficiently invade macrophages. The accessibility of IpaB was previously shown to increase upon removal of the G4C, and a further increase was observed for an O-Ag-deficient strain, indeed suggesting that the lipid A/core-linked O-Ag also contributes to shielding of the T3SS (10). The ability of the serotype 5a O-Ag synthesis and modification operon from S. flexneri to prevent the internalization of O-Ag-deficient S. sonnei indicates that the composition of the saccharides is not important for this phenotype. Furthermore, the inability of S. flexneri O-Ag when regulated by *wzzB* to complement for internalization of cell death suggests the modal length of the O-Ag is important. However, this strain also produced a small amount of O-Ag, and we cannot discount this as the reason for the failure to complement. Our data, and previously published data regarding the accessibility of the T3SS, support the conclusion that the O-Ag acts as a physical barrier to T3SS-mediated invasion rather than being antiphagocytic.

The results presented here, combined with previous investigations, indicate that S. sonnei and S. flexneri use different infection mechanisms. These mechanisms are also different from related Gram-negative enteric pathogens such as *Salmonella* spp. or enteropathogenic Escherichia coli, which also activate distinct inflammasome pathways in human macrophages (46–49). Increasing evidence points to S. sonnei being more adapted to an extracellular lifestyle since, compared to S. flexneri, it invades epithelial cells and macrophages poorly. This may partly explain the dominance of S. sonnei in developed countries, where improved living conditions, including reduced overcrowding and hence the person-to-person spread of pathogens, fails to lower S. sonnei infection rates. These studies highlight that further investigation into S. sonnei is required in order to implement appropriate measures to reduce infection rates.

## MATERIALS AND METHODS

### Bacterial strains and growth.

Unless otherwise stated, all *Shigella* strains (see Table S1 in the supplemental material) were routinely grown in tryptone soy broth (TSB) at 37°C with shaking at 200 rpm. Antibiotic selection was used when necessary as follows: 100 μg/ml ampicillin, 50 μg/ml kanamycin (Kn), 12.5 μg/ml chloramphenicol (Cm), 100 μg/ml erythromycin, 50 μg/ml streptomycin (Sm), and 10 μg/ml gentamicin (Gm).

10.1128/mBio.02654-19.4 TABLE S1 Strains used in this study. Download Table S1, DOCX file, 0.02 MB. Copyright © 2019 Watson et al. 2019 Watson et al. This content is distributed under the terms of the Creative Commons Attribution 4.0 International license.

### Cloning and mutagenesis.

S. sonnei LVP^Stabile^, Δ*waaL*, Δ*tssB*, and ΔO-Ag strains were constructed as follows (primer sequences are in Table S2 in the supplemental material). For S. sonnei LVP^Stabile^ nucleotides (nt) 82936 to 83715 and nt 83716 to 84215 were amplified using primers 1 and 2 and primers 3 and 4. The chloramphenicol cassette was amplified from pKD3 using primers 21 and 22. Overlapping PCR was used to construct the mutagenesis fragment 82936–83715-Cm-83716–84215; note that the P1-P2 fragment was inserted in the opposite orientation). This fragment was further amplified by PCR with primers 1 and 4. Then, 2 μg of PCR product was electroporated into S. sonnei 53G/pKD46 induced with 1 mM l-arabinose for 45 min to express Lambda Red recombinase genes. The electroporation was plated on TSB supplemented with Cm. Genomic insertion of *cat* was verified by PCR using primers 5 and 22.

10.1128/mBio.02654-19.5 TABLE S2 Primers used in this study. Download Table S2, DOCX file, 0.02 MB. Copyright © 2019 Watson et al. 2019 Watson et al. This content is distributed under the terms of the Creative Commons Attribution 4.0 International license.

For the S. sonnei Δ*tssB* mutant, 500-bp fragments flanking *tssB* were amplified using primers 6 and 7 and primers 8 and 9. The kanamycin cassette was amplified from pKD4 using primers 21 and 22. Overlapping PCR was used to construct the mutagenesis fragment consisting of 5′ *tssB*-Kn-3′ *tssB*. This fragment was further amplified by PCR with the primers 6 and 9. Then, 2 μg of PCR product was electroporated into S. sonnei 53G/pKD46 induced with 1 mM l-arabinose for 45 min to express Lambda Red recombinase genes. The electroporation was plated on TSB supplemented with Kn. Genomic insertion of *kan* was verified by PCR using the primers 10 and 22.

For the S. sonnei Δ*waaL* mutant, 500-bp fragments flanking *waaL* were amplified using primers 11 and 12 and primers 13 and 14. The kanamycin cassette was amplified from pKD4 using primers 21 and 22. Overlapping PCR was used to construct the mutagenesis fragment consisting of 5′ *waaL*-Kn-3′ *waaL*. This construct and pSEVA612S were digested with BamHI and EcoRI, ligated, and transformed into E. coli CC118-λ*pir*. The resulting plasmid, pSEVAΔwaaL-Kn, was conjugated into S. sonnei 53G. Briefly, 20 μl of helper E. coli 1047 pRK2013 was incubated for 2 h at 37°C with 20 μl of the donor strain (E. coli CC118-λ*pir* pSEVAΔwaaL) on Luria-Bertani (LB) agar. Then, 40 μl of the receiver strain (S. sonnei 53G/pACBSR) was added, and the plate was incubated for 4 h at 37°C. Conjugants were selected on TSB agar supplemented with Gm and Sm. Individual colonies were grown in TSB supplemented with Sm and 0.4% (wt/vol) l-arabinose (Sigma) for 8 h to induce expression of the I-SceI endonuclease from pACBSR and then plated on Kn plates. Genomic deletion of *waaL* was verified by PCR using primers 15 and 22. The strains were passaged several times in liquid TSB to remove pACBSR, and bacteria sensitive to Sm were selected.

S. sonnei ΔO-Ag mutant was constructed by amplifying 500-bp fragments upstream of *wbgT* and downstream of *wbgZ* using primers 16 and 17 and primers 18 and 19. The kanamycin cassette was amplified from pKD4 using primers 21 and 22. Overlapping PCR was used to construct the mutagenesis fragment consisting of 5′ *wbgT* -kan-3′ *wbgZ*. This construct and pSEVA612S were digested with BamHI and EcoRI, ligated, and transformed into E. coli CC118-λ*pir*. The resulting plasmid, pSEVAΔO-Ag-Kn, was conjugated into S. sonnei 53G, as described above. Genomic deletion of ΔO-Ag was verified by PCR using primers 20 and 22.

Complementation vectors were constructed using standard molecular biology techniques. The 53G O-Ag operon was amplified with primers 23 and 24. The PCR product and pSEVA471 were digested with BamHI and ligated to create pO-Ag^Ss^. The M90T *gtr* operon was amplified with primers 25 and 26. The PCR product and pSEVA471 were digested with KpnI and BamHI and ligated to create pSEVA471-gtr. The M90T O-Ag operon was amplified with primers 27 and 28. The PCR product and pSEVA471-gtr were digested with BamHI and XbaI and ligated to create pOAg^Sf5a^. The M90T *wzzB* gene was amplified with primers 29 and 30. The PCR product and pOAg^Sf5a^ were digested with KpnI and ligated to create pOAg^Sf5a/^*^wzzB^*. All complementation constructs include predicted promoters and terminators.

### Cell culture and infection.

THP-1 cells were maintained in Roswell Park Memorial Institute (RPMI) medium supplemented with 10% heat-inactivated fetal bovine serum (FBS), 5 mM HEPES, 5 mM sodium pyruvate, 100 μg/ml penicillin, and 100 μg/ml streptomycin. Cells were seeded at 7.5 × 10^5^ cells/ml 72 h prior to infection in complete RPMI plus 100 ng/ml PMA. At 24 h prior to infection, the medium was replaced with phenol-red free, PMA-free complete RPMI medium. HeLa cells were maintained in Dulbecco modified Eagle medium (1,000 mg/liter glucose) supplemented with 10% FBS. Cells were seeded at 1 × 10^5^ cells/ml 24 h prior to infection. All cell lines were incubated at 37°C and 5% CO_2_. Cells were infected with the indicated MOI and centrifuged for 10 min at 600 × *g* to synchronize infection. At 30 min postcentrifugation, Gm (150 μg/ml) was added directly to wells for the remainder of the experiment. Where indicated, inhibitors—Z-VAD-fmk (50 μM; R&D Systems) or MCC950 (5 μM; Tocris Bioscience)—were added to cells 1 h prior to infection. To induce NLRP3-driven caspase-1 activation, the cells were primed with ultrapure O111:B4 LPS (250 ng/ml; Invivogen) for 3 h and then treated with nigericin (20 μM; Sigma) for 45 min. To induce caspase-4 activation, unprimed cells were transfected with LPS (5 μg/ml) using Lipofectamine 2000 (1% [wt/vol]; Invitrogen).

Infected HeLa cells were washed and fixed in 2.5% glutaraldehyde for analysis by SEM at an accelerating voltage of 25 kV using a JEOL JSM‐5300 scanning electron microscope (JEOL, Herts, UK).

### Generation of cell lines.

The THP1 GSDMD^miR^ cell line was described previously (50); THP1 Casp1 knockout (KO), THP1 Casp4 KO, and THP1 ASC KO cells were all kindly provided by Veit Hornung (51).

### Isolation of primary hMDMs.

Leukocytes cones were obtained from the NHS blood and transfusion service (from anonymous healthy donations), as previously described (46). Blood from each donor was diluted 1:4 with phosphate-buffered saline (PBS), transferred into a LeucoSep tube (Greiner Bio-One), and centrifuged at 1,000 × *g* for 20 min at room temperature (slow acceleration and deceleration to prevent disturbance of the layers) to obtain the buffy coat containing white blood cells. This was separated and washed three times with RPMI. Cell were washed with MACS buffer (50 mg/ml bovine serum albumin [BSA] and 2 mM EDTA in PBS). CD14^+^ cells were isolated by MACS using biotinylated anti-CD14^+^ antibody and anti-biotin microbeads according to the manufacture’s protocol (Miltenyi Biotec). Monocytes were cultured in complete RPMI plus 20 ng/ml recombinant human macrophage colony-stimulating factor (M-CSF) for 7 days to promote differentiation into hMDMs. The medium was replaced with complete RPMI lacking antibiotics and M-CSF 24 h prior to infection.

### Internalization and vacuole escape assays.

To prevent cell death, cells were treated with Z-VAD-fmk (50 μM) 1 h prior to infection. The cells were infected with bacteria as described above. For internalization assays, the cells were washed with serum-free RPMI and lysed with TritonX-100 (0.5%) at 1.5 h postinfection. For vacuole escape assays, the cells were treated 30 min postinfection either with 200 μg/ml chloroquine and 150 μg/ml gentamicin or with 150 μg/ml gentamicin alone for 1 h and then lysed with Triton X-100 (0.5%). Serial dilutions were prepared, plated on LB agar, and incubated overnight at 37°C.

### PI uptake assays.

Cells and bacterial strains were prepared as described above. Prior to infection, the cells were supplemented with 5 μg/ml PI (Invitrogen). For time course assays, fluorescence was measured at 630 nm every 10 min with a POLARStar 623 Omega plate reader (BMG Labtech) (50). Uninfected controls treated with Triton X-100 (0.05%) were used to calculate the percent uptake.

### LDH assays.

Infections were performed as described above. At 3 h postinfection, supernatants were harvested. An LDH assay was performed according to the kit instructions (CytoTox 96 nonradioactive cytotoxicity assay; Promega). The absorbance was measured at 490 nm, and values are expressed as percentages of the 100% lysis control. All values are normalized to the uninfected control.

### Immunoblots.

Infections were performed as described previously, except that prior to infection the cells were washed with PBS and infections were done in Opti-MEM plus 5 mM sodium pyruvate. Supernatants were precipitated in acetone (1:4 [vol/vol]) overnight at –20°C, the acetone was aspirated, and the samples were left to air dry. Cells were lysed in radioimmunoprecipitation assay buffer (120 mM Tris [pH 8.0], 300 mM NaCl, 2% NP-40, 1% sodium deoxycholate, 2 mM EDTA) supplemented with complete protease inhibitor and 1 mM phenylmethylsulfonyl fluoride. Laemmli buffer and 5% 2-mercaptoethanol were added to the lysates. Precipitated supernatants were resuspended in respective cell lysates to create pooled samples. Mouse anti-hcaspase-1 (AdipoGen), mouse anti-caspase-4 (Santa Cruz Biotechnology), goat anti-hIL1β (R&D Systems), and rabbit anti-hIL18 (MBL International) were used at 1:1,000 dilutions, and mouse anti-hGSDMD (Santa Cruz Biotechnology) was used at a 1:500 dilution.

### Immunofluorescence microscopy.

Cells were seeded and infected as described previously. To calculate the percentage of THP1 cells infected, in/out staining was performed as follows. At 40 min after the addition of bacteria (*T* = 0), the cells were washed three times with cold PBS. Rabbit anti-sonnei (1:100; phase 1 and 2 sera; Fisher Scientific) or rabbit anti-flexneri (1:500; serotype 5a sera; PHE) diluted in 2% BSA–PBS was added to the cells. The cells were then incubated with antibodies on ice for 30 min. Next, the cells were washed with cold PBS and incubated on ice with donkey anti-rabbit-Alexa 594. (1:500; 2% BSA–PBS). Cells were fixed with 2% paraformaldehyde diluted in PBS for 20 min, washed in PBS, and neutralized with 50 mM NH_4_Cl. Then, 0.1% Triton X-100 was added to cells for 8 min to permeabilize them. Cells were incubated with DAPI (4′,6′-diamidino-2-phenylindole) (1:1,000; Invitrogen) and phalloidin Alexa 647 (1:100; Invitrogen) in 2% BSA-PBS. Coverslips were mounted onto slides with ProLong Gold antifade mountant and visualized using a Zeiss Axio Observer Z1 microscope. To count ASC foci, ASC^mRFP^ cells (46) were infected as described previously, washed in PBS at 3 h postinfection, and fixed. The protocol was then continued as described above.

### LPS preparation and visualization.

Crude LPS was prepared as follows. First, 1.5 ml of overnight culture was centrifuged, resuspended in Laemmli buffer, and boiled for 5 min. Then, proteinase K (1 mg/ml) was added, followed by incubation for 2 h at 56°C. Next, 2-mercaptoethanol (5%) was added, the samples were boiled for 5 min, and 5 μl of each sample was separated by 12% SDS-PAGE. The gel was either transferred to polyvinylidene difluoride and incubated with S. flexneri serotype 5a antibody (PHE) or S. sonnei phase I antibody (Abcam), followed by anti-rabbit horseradish peroxidase, and developed by chemiluminescence, or fixed and silver stained as previously described (52).

### Statistical analysis.

The number of independent repeats performed for each experiment was determined (indicated by “*n*” in the figure legends). One-way analysis of variance (ANOVA) or a Student *t* test was performed to compare means, as implemented in GraphPad Prism 8. Errors bars represent the standard errors of the means throughout.
