# Supplementary figures and images for: Shigella sonnei O-Antigen Inhibits Internalization, Vacuole Escape, and Inflammasome Activation
Source: mBio. 2019 Dec 17;10(6):e02654-19. doi: 10.1128/mBio.02654-19 (PMC6918081; doi:10.1128/mBio.02654-19)

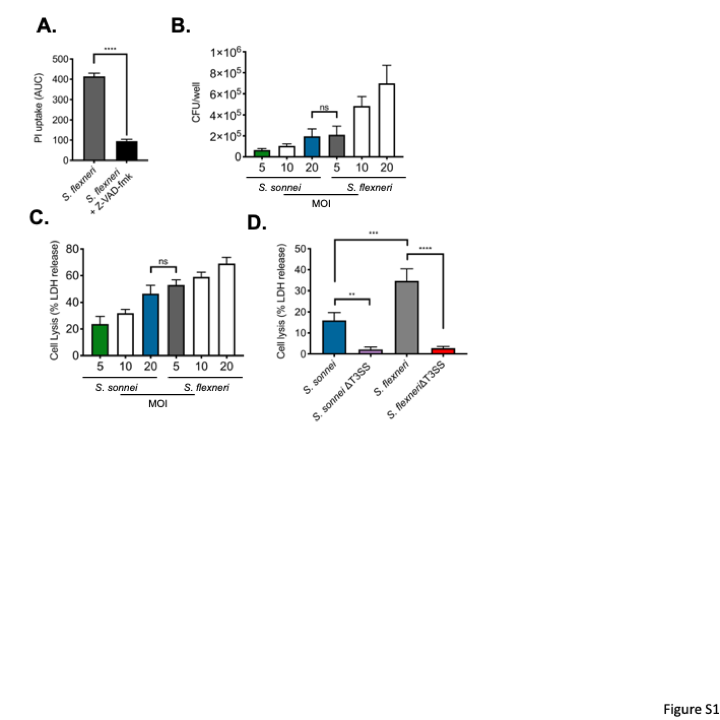

Supplement: FIG S1 [file mBio.02654-19-sf001.tif]

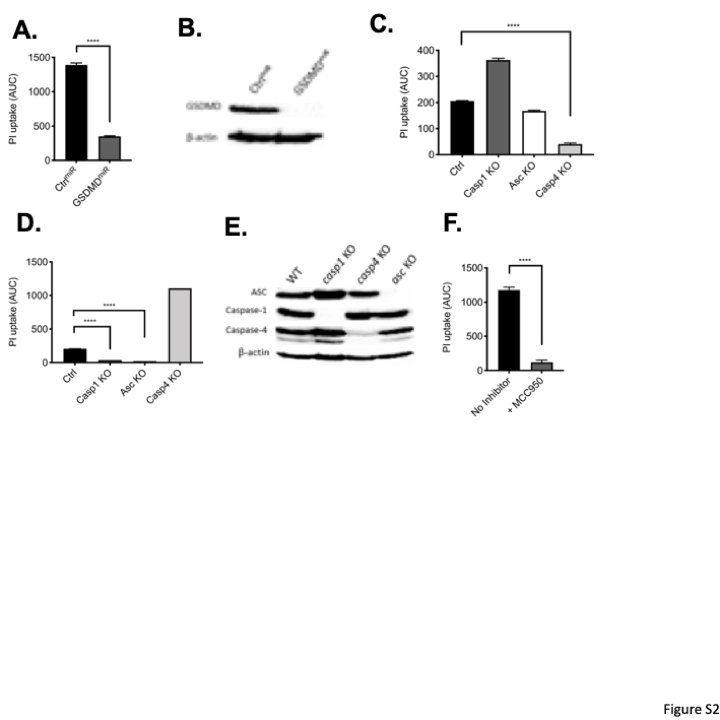

Supplement: FIG S2 [file mBio.02654-19-sf002.tif]

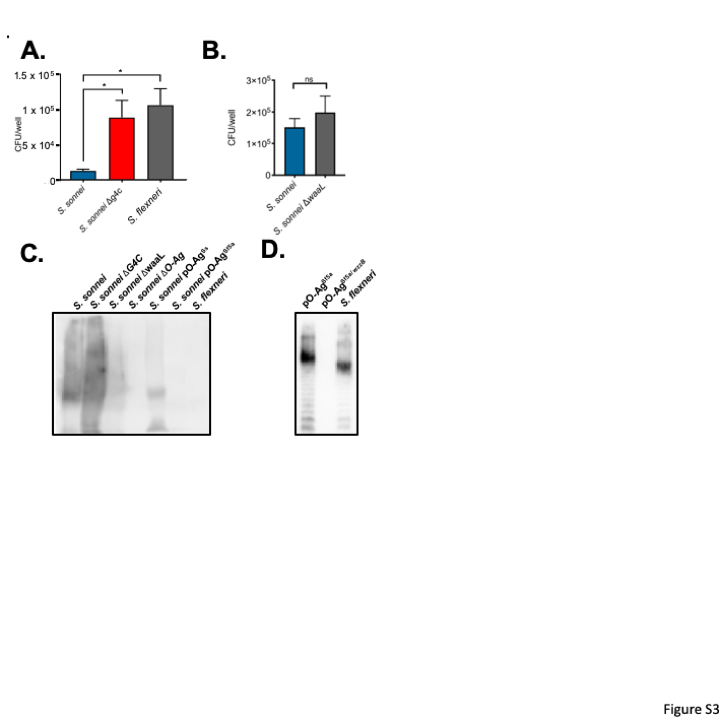

Supplement: FIG S3 [file mBio.02654-19-sf003.tif]
