# Supplementary material for: Shigella sonnei O-Antigen Inhibits Internalization, Vacuole Escape, and Inflammasome Activation
Source: mBio. 2019 Dec 17;10(6):e02654-19. doi: 10.1128/mBio.02654-19 (PMC6918081; doi:10.1128/mBio.02654-19)
Supplement: TABLE S1 [file mBio.02654-19-st001.docx]

Table S1: Strains used in this study

| Strain | Details | Source |
| --- | --- | --- |
| *S. sonnei* 53G |  | (54) |
| *S. sonnei* 381 | Clinical isolate H140860381 | (55) |
| S. *flexneri* M90T | Serotype 5a | (56) |
| *S. flexneri* 2457T | Serotype 2a | (57) |
| *S. sonnei* ΔT3SS | *mxiD* replaced with *aphA-3,* conferring kanamycin resistance | (55) |
| *S. flexneri* ΔT3SS | mxiD replaced with aphA-3, conferring kanamycin resistance | (55) |
| *S. sonnei* ΔG4C | *g4c* cluster (genes *ymcDCBA, yccZ, etp*, *etk*) replaced with *erm* cassette conferring resistance to erythromycin | A kind gift from C. Gerke (10) |
| *E. coli* helper | *E. coli* 1047 harbouring pRK2013 which encodes RK2 transfer genes | (58) |
| *E. coli* CC118-λpir | Expresses λ protein from a lysogenic phage | (59) |
| *S. sonnei* 53G receiver | *S. sonnei* 53G harbouring pACBSR which encodes arabinose inducible lamba red recombinase and I-SceI endonuclease | (60) |
| *S. sonnei* LVP^Stabile^ | *S. sonnei* 53G with *cat* (conferring chloramphenicol resistance) inserted at nt 83716 of 53G LVP, allowing selection of LVP positive strains |  |
| *S. sonnei* Δ*waaL* | *S. sonnei* 53G with *waaL* replaced with *aphA-3,* conferring kanamycin resistance | This study |
| *S. sonnei* ΔO-Ag | *S. sonnei* 53G with O-Ag biosynthesis operon (*wbgTU, wzx, wzy,* IS630, *wbgVWXYZ)* replaced with *aphA-3*, conferring kanamycin resistance | This study |
| *S. sonnei* Δ*tssB* | *S. sonnei* 53G with *tssV* replaced with *aphA-3,* conferring kanamycin resistance | This study |
| *S. sonnei*  ΔO-Ag + pO-Ag^Ss^ | O-Ag biosynthesis operon (*wzz, wbgTU, wzx, wzy,* IS630, *wbgVWXYZ* with 102 bp 5’ of *wzz* and 76 bp 3’ of *wbgZ*) from 53G inserted into pSEVA471:SmR expressed in a *S. sonnei* 53G ΔOAg background | This study |
| *S. sonnei* 53G ΔO-Ag + pO-Ag^Sf5a^ | *gtr* modification (*gtrABV* with 100 bp 5’ of *gtrA* and 100 bp 3’ of *gtrV*) and O-Ag biosynthesis (*rfbBDACEFG, rfc, rfbIJ*, orf, orf with 500 bp 5’ of *rfbB* and 200 bp 3’ of orf) operons from M90T inserted into pSEVA471:SmR expressed in a *S. sonnei* 53G ΔOAg background | This study |
| *S. sonnei* 53G ΔO-Ag + pO-Ag^Sf5a/^*^wzzB^* | *wzzB* (202bp 5’ and 101 bp 3’ of *wzzB*) from M90T inserted into pO-Ag^Sf5a^ expressed in a *S. sonnei* 53G ΔOAg background | This study |
