# Supplementary material for: Shigella sonnei O-Antigen Inhibits Internalization, Vacuole Escape, and Inflammasome Activation
Source: mBio. 2019 Dec 17;10(6):e02654-19. doi: 10.1128/mBio.02654-19 (PMC6918081; doi:10.1128/mBio.02654-19)
Supplement: TABLE S2 [file mBio.02654-19-st002.docx]

**Table S2:** Primers used in this study.

| No. | Name | Sequence (Restriction enzyme sites are underlined) |
| --- | --- | --- |
| 1 | 53G.nt82936.F | ATGTCGGAACAACGGAAG |
| 2 | 53G.nt83715.p2.R | CTAAGGAGGATATTCATATGCTTACAAAGCCTCATTAG |
| 3 | 53G.nt83716.p1.F | GCAGCTCCAGCCTACACAATTGTCGTTTTTTATCTGTG |
| 4 | 53G.nt84215.R | CTGTTTTATTCTCCTAATCTTC |
| 5 | 53G.nt84574.R | GCTATCTGTTATTCAATCC |
| 6 | 53G.492bpus*tssB*.F | CTGGATAACCATACCCAAC |
| 7 | 53G*.tssB*.K.R | GCAGCTCCAGCCTACACAGCTGCCTTCAAATTTTTTGC |
| 8 | 53G.*tssB*.K.F | CTAAGGAGGATATTCATATGCCACCACTATTTCATTAAAC |
| 9 | 53G.422bpds*tssB*.R | CATGCTGTCCACGGCCAG |
| 10 | 53G.1786bpds*tssB*.F | GTTACTCCCTGTCTAACC |
| 11 | 53G.500bpus*waaL*.BamHI.F | ACGGATCCAAGATTTACGACTAGGAGAAG |
| 12 | 53G.500bpus*waaL*.p1.R | GAAGCAGCTCCAGCCTACACAATAGTTTCAACCTATGCTAC |
| 13 | 53G.500bpds*waaL*.p2.F | CTAAGGAGGATATTCATATGTGAATATGTGAAATAAAATCAGC |
| 14 | 53G.500bpds*waaL*.EcoRI.R | GCTGAATTCCCGTATTTCTTCATGCGAC |
| 15 | 53G.*waaC*.F | GAGCAATGATTACCCTTTAG |
| 16 | 53G.500bpus*wbgT*.BamH1.F | CTAGGATCCAGTCTTGATGAGTTTGGTC |
| 17 | 53G.500bpuswbgT.p1.R | GAAGCAGCTCCAGCCTACACACTATTTCGCATTGACACAAC |
| 18 | 53G.500bpdswbgZ.p2.F | CTAAGGAGGATATTCATATGTTGCTTAGGTTTAACCTTCC |
| 19 | 53G.500bpdswbgZ.EcoRI.R | CTAGAATTCGTGAATGGAGGCCATAAC |
| 20 | 53G.wzz.F | GCACCACGTATTGCTGAATTAG |
| 21 | P1 | TGTGTAGGCTGGAGCTGCTTC |
| 22 | P2 | CATATGAATATCCTCCTTAG |
| 23 | 53G OAg.BamHI.F | CTAGGATCCCAGCGCTTTGGGAGCTGAAAC |
| 24 | 53G OAg.BamHI.R | TAGGGATCCGGCGGTCACGATGTACCG |
| 25 | M90T gtroperon.KpnI.F | CTAGGTACCCTTATGTGTCTCAGTTTTGTCTCATCAG |
| 26 | M90T gtroperon.BamHI.R | GTGGGATCCAGAGGCCGTTAATTATAACTGCAGATG |
| 27 | M90T OAg.BamHI.F | CTAGGATCCTGACCGGCGACAGTTACG |
| 28 | M90T OAg.XbaI.R | AGATCTAGACAGAGATCCAAAAACTACGGACATAC |
| 29 | M90T wzzB.KpnI.F | CTAGGTACCCAAGCCGACGTCATTATCTC |
| 30 | M90T wzzB.KpnI.R | CTAGGTACCCAACACACGTAGGCCGGATAAG |
